# Investigating the potential of proton therapy for hypoxia-targeted dose escalation in non-small cell lung cancer

**DOI:** 10.1186/s13014-021-01914-2

**Published:** 2021-10-11

**Authors:** Andreas Köthe, Nicola Bizzocchi, Sairos Safai, Antony John Lomax, Damien Charles Weber, Giovanni Fattori

**Affiliations:** 1 Center for Proton Therapy, Paul Scherrer Institute, 5232 Villigen, Switzerland; 2 Department of Physics, ETH-Hönggerberg, Zurich, Switzerland; 3 Radiation Oncology Department, Inselspital Universitätsspital Bern, Bern, Switzerland; 4 Radiation Oncology Department, University Hospital of Zurich, Zurich, Switzerland

**Keywords:** NSCLC, Proton therapy, Tumor hypoxia, PET

## Abstract

**Background:**

Hypoxia is known to be prevalent in solid tumors such as non-small cell lung cancer (NSCLC) and reportedly correlates with poor prognostic clinical outcome. PET imaging can provide in-vivo hypoxia measurements to support targeted radiotherapy treatment planning. We explore the potential of proton therapy in performing patient-specific dose escalation and compare it with photon volumetric modulated arc therapy (VMAT).

**Methods:**

Dose escalation has been calibrated to the patient specific tumor response of ten stage IIb-IIIb NSCLC patients by combining HX4-PET imaging and radiobiological modelling of oxygen enhancement ratio (OER) to target variable tumor hypoxia. In a dose-escalation-by-contour approach, escalated dose levels were simulated to the most hypoxic region of the primary target and its effectiveness in improving loco-regional tumor control was assessed. Furthermore, the impact on normal tissue of proton treatments including dose escalation was evaluated in comparison to the normal tissue complication probability (NTCP) of conventional VMAT plans.

**Results:**

Ignoring regions of tumor hypoxia can cause overestimation of TCP values by up to 10%, which can effectively be recovered on average to within 0.9% of the nominal TCP, using patient-specific dose escalations of up to 22% of the prescribed dose to PET defined hypoxic regions. Despite such dose escalations, the use of protons could also simultaneously reduce mean doses to the heart (− 14.3 Gy_RBE_), lung (− 8.3 Gy_RBE_), esophagus (− 6.9 Gy_RBE_) and spinal cord (− 3.8 Gy) compared to non-escalated VMAT plans. These reductions are predicted to lead to clinically relevant decreases in NTCP for radiation-induced pneumonitis (− 11.3%), high grade heart toxicity (− 7.4%) and esophagitis (− 7.5%).

**Conclusions:**

This study suggests that the administration of proton therapy for dose escalation to patient specific regions of tumor hypoxia in the treatment of NSCLC can mitigate TCP reduction due to hypoxia-induced radio resistance, while simultaneously reducing NTCP levels even when compared to non-escalated treatments delivered with state-of-the-art photon techniques.

**Supplementary Information:**

The online version contains supplementary material available at 10.1186/s13014-021-01914-2.

## Background

Lung cancer is the most common type of cancer worldwide, with more than 2 million new cases a year and almost 1.8 million deaths [1]. Of these, 80% are non-small cell lung cancer (NSCLC) patients [2], typically treated with surgery and adjuvant treatment if needed or a combination of radio- and chemotherapy for advanced stages of disease. Hypoxia is known to be prevalent in large solid tumors and reportedly correlated with poor prognostic outcome [3], therefore assessment of hypoxia using PET imaging could be of great value in the management of NSCLC [4, 5]. Even though PET tracers, such as F-MISO, F-FAZA or F-HX4 have a demonstrated sensitivity to tissue oxygenation [6], hypoxia imaging is rarely part of radiotherapy routine imaging. That is partly due to the uncertainty in quantitative assessment and reproducibility of imaging biomarkers, but also due to the lack of standardized care pathways capable of making use of such additional information. A way to overcome hypoxia is dose escalation, an approach that has been tested in a few randomized trials, including NSCLC patients cohorts [7], albeit with questionable success. Photon therapy, as shown in the seminal RTOG 0617 trial [7], however might not be particularly suited for precise dose painting as required for hypoxia targeting, as the increase in integral dose in normal tissues increases the risk of toxicity. As such, proton therapy could be a better alternative, with dosimetric benefits for lung cancer treatments regarding normal tissue toxicity [8] as well as promising clinical results [9, 10] although the MDACC trial questioned the clinical benefit of protons delivered with a passive delivery paradigm with no motion-mitigation strategy other than increasing tumor margins [11]. A phase III trial (RTOG 1308; NCT01993810) [12] has randomized 2/3 of the accrual target and should give an answer to the value of protons for the treatment of these challenging patients. Moreover, image guidance [13] and motion management technologies [14] in pencil beam scanning (PBS) are continuously being refined and nowadays provide the technical background for the locoregional irradiation of hypoxic volumes of the tumor even in the presence of organ motion, should it be proven beneficial in terms of tumor control. However, to our knowledge, the potential for proton therapy treatments that include locally boosting hypoxic regions of NSCLC tumors has not yet been studied.

In this work we have explored a straight-forward approach to integrate hypoxia imaging in proton therapy planning as the basis to calibrate dose escalation for patient specific tumor response. We wished to evaluate the potential of proton therapy for such a treatment approach at different clinical levels assessing (i) what the effect of the proposed dose escalation strategy on tumor control probability (TCP) is, taking into account hypoxia-induced radio resistance and (ii) how doses to organs-at-risk (OARs) and the resulting normal tissue complication probabilities (NTCP) are affected by proton dose escalation compared to state-of-the-art photon treatments. We answered these questions by evaluating comparative treatments of 10 advanced-stage NSCLC patients to estimate potential benefits of proton therapy in the context of hypoxia-targeted dose escalation using TCP and a range of different NTCP models.

## Methods

### Patient data

Ten stage IIb-IIIb NSCLC patients with hypoxic volumes in the primary target have been selected from an ongoing phase II randomized clinical trial cohort (NCT01024829). The dataset, which is made freely available by Even et al. [15], includes for each patient the mid-ventilation planning CT with clinical structures drawn by experienced radio-oncologists, together with a HX4 PET image and associated low-dose CT obtained within a week of the planning CT acquisition. Relevant details of this cohort, including patient sex, age and tumor characteristics are summarized in Table 1. Patients with stage III upwards received concurrent chemotherapy.

**Table 1:** Clinical characteristics of the patients included in this study

Patient #	Sex	Age (years)	cTNM	UICC Stage	GTV Volume (total (primary)) [cc]
1	M	66	T2N3M0	IIIb	95.2 (78.6)
2	F	46	T2N2M0	IIIa	191.1 (169.5)
3	M	65	T3N2M0	IIIa	190.1 (148.8)
4	M	64	T4N2M0	IIIb	230.2 (214.3)
5	F	65	T2N2M0	IIIa	86 (32.8)
6	M	66	T3N2M0	IIIa	107.4 (95.3)
7	M	60	T4N1M0	IIIb	862.4 (853.6)
8	M	71	T2N3M0	IIIb	97.3 (42.8)
9	M	77	T3N2M0	IIIa	304.9 (296.3)
10	M	82	T3N0M0	IIb	63.3 (63.3)

### Treatment planning

For each patient, a conventional photon volumetric modulated arc therapy (VMAT) and pencil beam scanning proton (PBSPT) treatment were planned according to the criteria of the RTOG 1308 phase III randomized trial (NCT01993810) comparing photon vs. proton chemoradiotherapy for stage II-IIIb NSCLC patients. An overview of the planning constraints following the amendments from March 2018 is given in the Additional file 1: Supplementary Material S1. Nominal prescription dose to the planning target volume (PTV) is 70 Gy_RBE_, with the option to reduce it to 60 Gy_RBE_ should normal tissue constraints not be met. Patients undergo radiation therapy 5 days per week for a total of 35 fractions at 2 Gy_RBE_ per fraction. Primary gross tumor volumes (GTV_prim_; Table 1) and lymph nodes (GTV_lnx_) from Even et al*.* [15] were drawn on the mid-ventilation phase of a 4D FDG-PET/CT and expanded isotropically by 5 mm to create the clinical target volumes CTV_prim_ and CTV_Inx_. A uniform 5 mm PTV margin was applied to the lymph nodes. Our retrospective analysis of the primary target structures indicated that a CTV to PTV margin of the order of 10 mm was adjusted on a patient-by-patient basis accounting for breathing motion. The considered organs at risk (OARs) were the esophagus, spinal cord, heart, brachial plexus, as well as both lungs excluding the GTVs.

VMAT treatments were planned with RapidArc (Eclipse version 13, Varian Medical Systems, Palo Alto, CA, USA) with 2–3 full arcs for medial tumors and half arcs for the more lateral ones, using standard 6 MV machine data for a Varian True Beam. For the proton plans, an in-house treatment planning system (PSIPlan [16]) has been used to simulate proton treatments using beam data from Gantry 2 at our institute. Posterior or anterior beam arrangements were favored, with 3 fields individually optimized in single field optimization (SFO) mode using an RBE of 1.1 [17], which is standard practice in our clinical planning. An experienced treatment planner (NB) for both VMAT and PBSPT has reviewed and approved all the plans used in this study. Note, hypoxia guided dose escalation was performed only for the proton plans, as even without dose-escalation, all VMAT plans were close to the specified dose constraints, and dose escalation could not be performed without violating these.

### Hypoxia-guided dose escalation

In addition to standard photon and proton treatment plans, for each patient a dose escalated plan was created for proton therapy. HX4 PET hypoxia images were used in combination with calibrated oxygen enhancement ratio (OER) models to plan a localized dose boost to a sub-volume of the GTV. For each patient, the hypoxic region within the primary tumor (GTV_hypoxic_) was identified on PET images including the set of voxels with a tumor-to-background HX4 uptake ratio above 1.4 with respect to the aortic arch [15]. HX4 PET data were fused with the mid-ventilation planning CT via rigid registration of the low-dose CT associated with the PET acquisition. A first rough alignment of the bony anatomy was further refined with automatic registration using a region of interest on the primary target (Velocity 4.1, Varian, Palo Alto, CA, USA) and the resulting transformation used to propagate the GTV_hypoxic_ structure onto the planning images. Finally, to optimize dose homogeneity inside the GTV_hypoxic_, the structure was expanded by 3 mm in the planning process. Afterwards, PET standardized uptake was converted into partial oxygen pressure (pO_2_) following a HX4 to pO_2_ calibration from Ureba et al. [18] and based on that the OER calculated using the proton specific model proposed by Dahle et al. [19]. This conversion requires a definition of normoxic partial oxygen pressure, for which we have considered 30 mmHg. A uniform dose escalation factor (DE) for GTV_hypoxic_ was then defined as:

$$DE := \frac{OE{R}_{hypoxic}}{OE{R}_{NSCLC}}$$

where $OE{R}_{NSCLC}$ is an average value for NSCLC tumors which have an estimated mean pO_2_ equal to 16.4 mmHg [20]. This corresponds to a mean OER of 1.05, which is assumed to be accounted for in the treatment protocol and empirical definition of the prescription dose. OER_hypoxic_ is the OER in the hypoxic region of the tumor calculated based on the mean HX4 PET standardize uptake value (SUV) in the structure. At this stage, the OER was calculated with a constant proton linear energy transfer (LET) of 2 keV/µm, thus allowing optimization of plans in a wide range of clinical treatment planning systems that, most often, do not yet include sophisticated LET-based modelling. However, the implications of this assumption on tumor control are assessed by considering variable dose-averaged LET (LET_d_) in our evaluation of the results. Following the DE determination, a ‘dose-painting by contour’ approach was chosen to re-optimize the treatment plans for dose escalation, boosting the prescription dose (simultaneous integrated boost) to the GTV_hypoxic_ by the individual DE factor calculated for each patient in this volume. The same planning constraints as in the conventional plans were used with the addition of V_70 Gy*DE_ = 100% to GTV_hypoxic_.

### Estimation of tumor control

In order to estimate the effectiveness of dose escalation on tumor control, we compared tumor control probability (TCP) resulting from conventional and dose escalated proton plans. The probability of local tumor control was estimated according to an equivalent uniform dose (EUD)-based model [21] using a parametrization built on data from Okunieff et al. [22] which was developed on a multi-institutional cohort of NSCLC patients. Both voxel level evaluation as well as overall TCP for primary CTV were calculated using the EUD with volume parameter *a* = − 10 [23]. Reference TCP estimates of conventional and dose-escalated treatments calculated without knowledge of tissue oxygenation were reassessed accounting for the tumour OER of individual patients. Again, a baseline OER of 1.05 was assumed within the GTV_hypoxic_. Unlike in planning, however, voxel-wise dose-weighted LET_d_ has been taken into account for the evaluation of the dose distributions and assessment of TCP. With the primary intent of understanding the effects of hypoxia in different planning strategy scenarios we decoupled OER and RBE calculations by assuming a constant RBE of 1.1, while adapting the OER inside the primary target based on local oxygenation and variable LET_d_ (similarly to what was proposed in Dahle et al. [19]). Physical doses were transformed to RBE_1.1_-weighted doses and then, scaled by the OER inside the PTV prior to EUD calculation. This procedure, requiring voxel level information, has required the resampling of dose data and PET images to the resolution of the planning CT for which we have used trilinear interpolation (Velocity 4.1, Varian, Palo Alto, CA, USA).

### Estimation of normal tissue complication

The potential increase in healthy tissue toxicity due to dose escalation was assessed by comparing NTCP estimates with conventional, non-escalated photon therapy treatments for radiation pneumonitis [24, 25], radiation-induced esophagitis [26–28] and death due to heart failure [29]. In selecting the models, focus was put on externally validated models built on NSCLC cohorts. To avoid compromising the estimation of toxicity and thus our conclusions with the choice of organ architecture (serial/parallel), multiple models for pneumonitis and esophagitis were considered, providing results for various hypotheses. An overview of the employed models is given in Table 2. For more details the reader is referred to the Additional file 1: Supplementary Material S2. As the healthy tissue around the tumor was most likely not subject to abnormal oxygen level, photon dose and RBE_1.1_-weighted proton dose were used for NTCP calculations. Data analysis and statistical testing was performed in MATLAB (v2018b, The MathWorks, Natick, MA, USA). Statistical significance threshold was set to 0.05.

**Table 2:** Overview of applied TCP and NTCP models

Model	Type	Endpoint	References
TCP	EUD	Tumor control	Okunieff et al. [22], Chaikh and Balosso [23]
NTCP	Sigmoid	Symptomatic radiation pneumonitis	Appelt et al. [24]
NTCP	LKB	Radiation pneumonitis (grade ≥ 2)	Tucker et al. [25]
NTCP	Logistic	Severe acute esophagitis (grade ≥ 2)	Huang et al. [26]
NTCP	Logistic	Acute esophageal toxicity (grade ≥ 2)	Wijsman et al. [27]
NTCP	LKB	Radiation induced esophagitis (grade ≥ 2)	Wang et al. [28]
NTCP	Relative seriality	Death due to heart failure	Gagliardi et al. [29]

One TCP and six NTCP models were chosen based on multi-institutional cohorts, external validation and treatment modality. LKB: Lyman-Kutcher-Burman model, EUD: Equivalent uniform dose model

## Results

### Treatment plans, hypoxia and LET

All patients have been successfully planned to RTOG 1308 standard although prescription dose for patients 1 and 8 (20% of the cohort) had to be reduced to 60 Gy in order to meet the lung constraints for the photon plans. To keep the plans normalized to target coverage, proton prescriptions for these two patients were similarly reduced to 60 Gy, even though 70 Gy could have been achieved within constraints. A gallery of dose distributions with photon, proton and dose escalated proton plans for exemplary cases can be found in the Additional file 1: Supplementary Material S3. The overview of prescription dose, volume of the hypoxic structure GTV_hypoxic_, LET_d_, pO_2_ as well as OER estimations within and outside of the hypoxic tumor volume for each patient is given in Table 3. DE factors ranged from 1.07 to 1.22 (median, 1.12) for boost volumes between 5.2 and 58.9 cc (median, 30.4). The boost factor is systematically lower than the estimated OER in the hypoxic region (median 1.16) due to our assumption of a baseline OER within GTV_hypoxic_ reducing the need of dose escalation by about 5%. Median partial oxygen pressure in the hypoxic volume was in the 5 to 9.2 mmHg range (median 6.95 mmHg) and between 28 and 30 mmHg in the volume of the primary CTV that was not considered to be hypoxic. LET_d_ calculations showed median LET_d_ values between 1.9 and 2.6 keV/µm (median 2.2) in the primary CTV for the dose escalated proton plans, which is only marginally higher than our assumption of a constant 2 keV/µm used for the DE factor estimation. LET_d_ values in the target were negligibly lower (< 0.1 keV/µm) for the conventional proton plans compared to the dose escalated ones. Exemplary LET_d_ distributions and LET_d_ differences between conventional and dose escalated proton plans can be found in the Additional file 1: Supplementary Material S4.

**Table 3:** Overview over quantities related to the effect of hypoxia in the primary CTV and hypoxic volume for each patient

Patient	Dose to PTV [Gy_RBE_]	Volume of GTV_hypoxic_ [cc]	DE	LET_d_ in CTV_prim_ [keV/µm]	pO_2_ in GTV_hypoxic_ [mmHg]	pO_2_ in CTV_prim_ – GTV_hypoxic_ [mmHg]	OER in GTV_hypoxic_	OER in CTV_prim_-GTV_hypoxic_
1	60	48.3	1.17	2.2 (2.0,2.4)	5.4 (4.5,6.6)	29.5 (12.9,30)	1.22 (1.18,1.27)	1.0 (1.0,1.08)
2	70	58.9	1.15	2.3 (2.2,2.6)	6.0 (5.3,6.9)	25.1 (11.9,30)	1.2 (1.17,1.23)	1.0 (1.0,1.08)
3	70	19.8	1.10	1.9 (1.6,2.3)	7.9 (7.0,8.7)	30.0 (23.3,30)	1.15 (1.13,1.17)	1.0 (1.0,1.02)
4	70	37.5	1.13	2.1 (1.9,2.4)	6.6 (6.0,7.2)	30.0 (15.7,30)	1.18 (1.16,1.2)	1.0 (1.0,1.05)
5	70	8.0	1.12	2.6 (2.4,2.9)	7.2 (6.1,8.4)	30.0 (19.0,30)	1.16 (1.13,1.2)	1.0 (1.0,1.03)
6	70	52	1.22	2.5 (2.0,3.0)	5.0 (3.5,6.4)	29.2 (14.3,30)	1.24 (1.19,1.34)	1.0 (1.0,1.06)
7	70	46.8	1.10	1.9 (1.7,2.1)	8.2 (6.8,9.3)	30.0 (30,30)	1.14 (1.12,1.17)	1.0 (1.0,1.0)
8	60	23.3	1.07	2.2 (2.0,2.4)	9.2 (8.3,10.5)	30.0 (30,30)	1.12 (1.1,1.14)	1.0 (1.0,1.0)
9	70	20.9	1.13	2.2 (1.9,2.6)	6.7 (6.2,7.2)	28.0 (12.7,30)	1.18 (1.16,1.19)	1.0 (1.0,1.07)
10	70	5.2	1.12	2.6 (2.2,3.2)	7.4 (6.3,8.4)	30.0 (19.9,30)	1.16 (1.13,1.19)	1.0 (1.0,1.03)
Cohort Median	70	30.4	1.13	2.2	7	30	1.17	1

For LET_d_, pO2 and OER values, the median is reported with the 25 and 75 percentiles indicated in brackets. While the normoxic part of the CTV resulted in an OER of 1, the decreased levels of oxygen within the GTV_hypoxic_ led to increased OER, thus higher amounts of dose necessary to control the tumour

### Tumor control probability

**Fig. 1:**
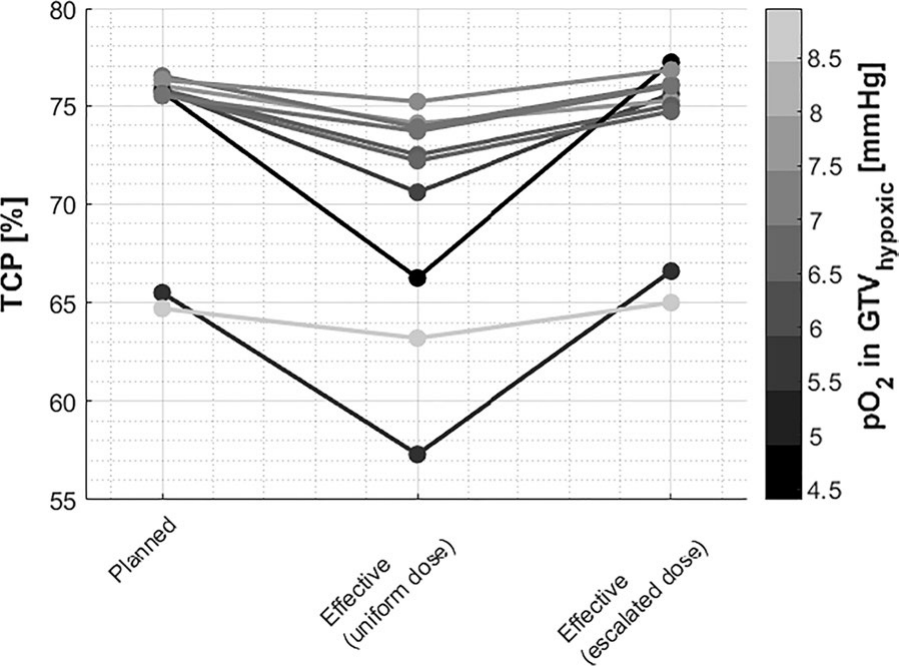
TCP for each patient in the cohort calculated for the primary CTV. Losses in TCP due to hypoxia are shown in the reduced effective TCPs for the uniform dose prescription proton plans. Escalating the dose restores the TCP to what was originally planned for. The loss in effective TCP for uniform dose plans compared to the initial plan was correlated to the degree of hypoxia in the GTV_hypoxic_ (grayscale colorbar)

TCP of conventional plans has been reassessed accounting for variable OER and the possible inclusion of dose escalation. Effective TCPs, calculated taking into account the increased OER due to hypoxia, were generally lower than their estimates neglecting the actual tissue oxygenation. The amount of TCP reduction was primarily a function of the specific tumour hypoxia (Fig. 1 – Planned vs. Effective (uniform dose)) and ranged from 1.1% (mean pO_2_ in GTV_hypoxic_ = 9 mmHg, patient 8), up to 9.5% (mean pO_2_ = 4.4 mmHg, patient 6). Despite the large inter-subject variability, the TCP was overestimated in our cohort by an average of 3.85% when OER effects were neglected. This hypoxia-induced reduction in local control was however effectively compensated for by dose escalation, a strategy capable of narrowing the discrepancy with the planned TCP down to 0.9% on average.

**Fig. 2:**
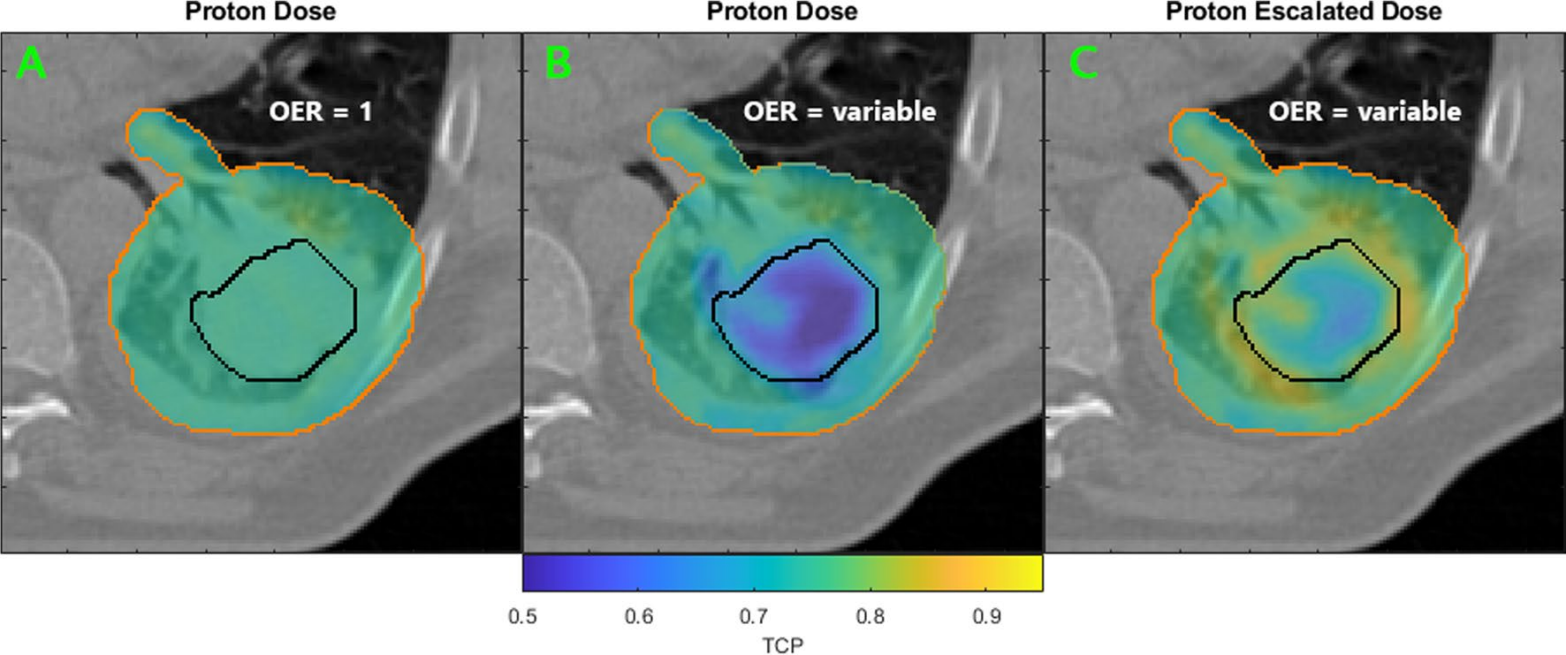
Exemplary voxel-wise TCP calculation from Patient 6 for the conventional proton plan with uniform dose prescription (**A**,**B**) and patient-specific escalated dose (22%) to the hypoxic tumor volume (**C**). PTV contours in orange, the hypoxic region GTV_hypoxic_ in black. Taking into account hypoxia information and its influence on TCP, locoregional losses can be observed (**B**) compared to the planned TCP (**A**) where OER is assumed to be consistently 1 throughout the target. The dose escalation (**C**) counteracts the increased radioresistance caused by hypoxia by increasing the dose to the radioresistant area and thus recovers TCP

The impact of hypoxia on voxel-wise TCP is shown in Fig. 2 for the primary PTV of the patient with the most severe hypoxia (Patient 6, 22% dose escalation). As a consequence of the homogeneous dose escalation approach, TCP was restored in the hypoxic region of the target (within the black contour) and improved beyond its initial value (OER = 1) in the surrounding tissues, included or in close proximity of the GTV_hypoxic_ planning margin.

### Normal tissue toxicity

**Fig. 3:**
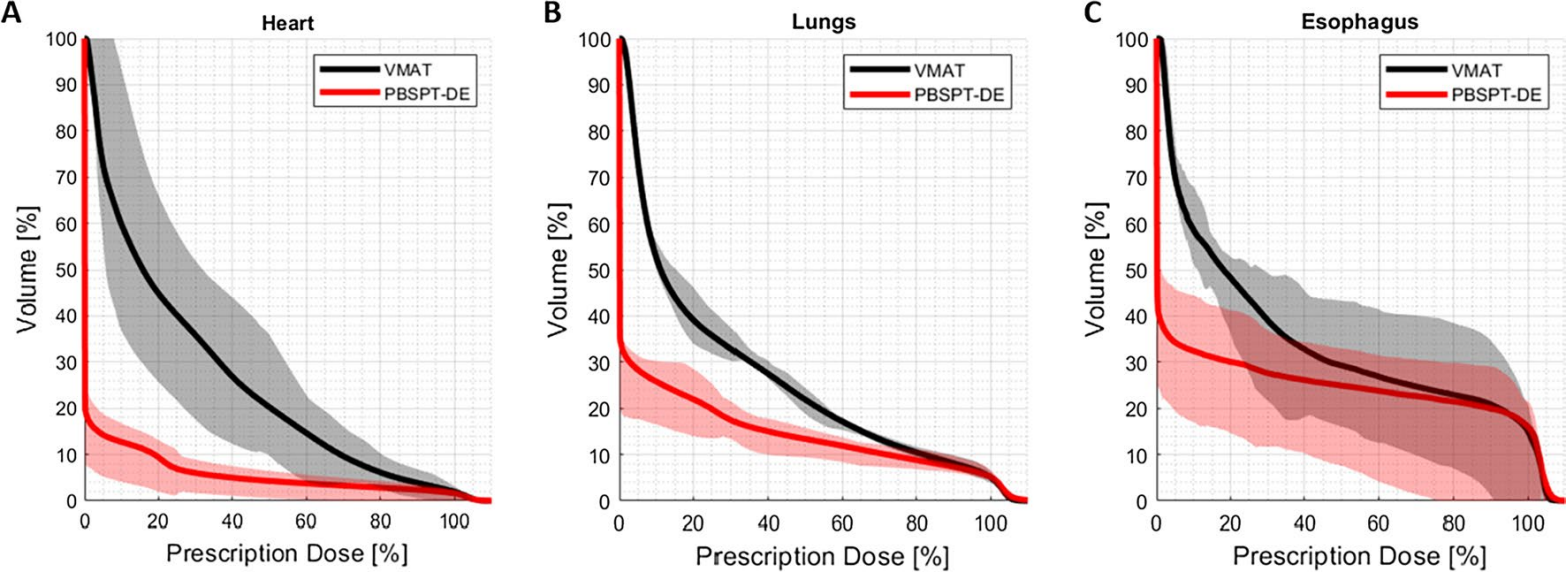
Mean DVHs for heart, lungs and esophagus are shown for the whole cohort (solid lines) for conventional non-escalated VMAT plans and dose-escalated proton plans. Color bands represent the cohort 25th to 75th percentile. Reductions in dose with dose-escalated proton plans are clearly visible for all OARs, despite the increased dose to the target

The positive impact of dose escalation on TCP has to be weighed against the increase of the risk of radiation-induced toxicities. As such, the dose to organs at risk for the most unfavorable proton plans, those that included dose escalation, were compared with conventional photon treatments. Mean dose-volume histograms for lungs, heart and esophagus show significantly lower doses to OARs for proton treatments compared to the conventional VMAT plans (Fig. 3). Substantial mean dose reductions for protons could be observed across the whole cohort simulating the whole treatment course: on average the heart received 14.3 Gy less dose, the lungs 8.3 Gy, the esophagus 6.9 Gy and the spinal cord 3.8 Gy. As can be furthermore seen in Fig. 3, the OAR constraints were already challenging to be met with non-escalated photon treatments, while proton doses were considerably below the constraints (e.g. Lungs V_20 Gy_ < 37% or Heart V_30 Gy_ < 50%).

**Fig. 4:**
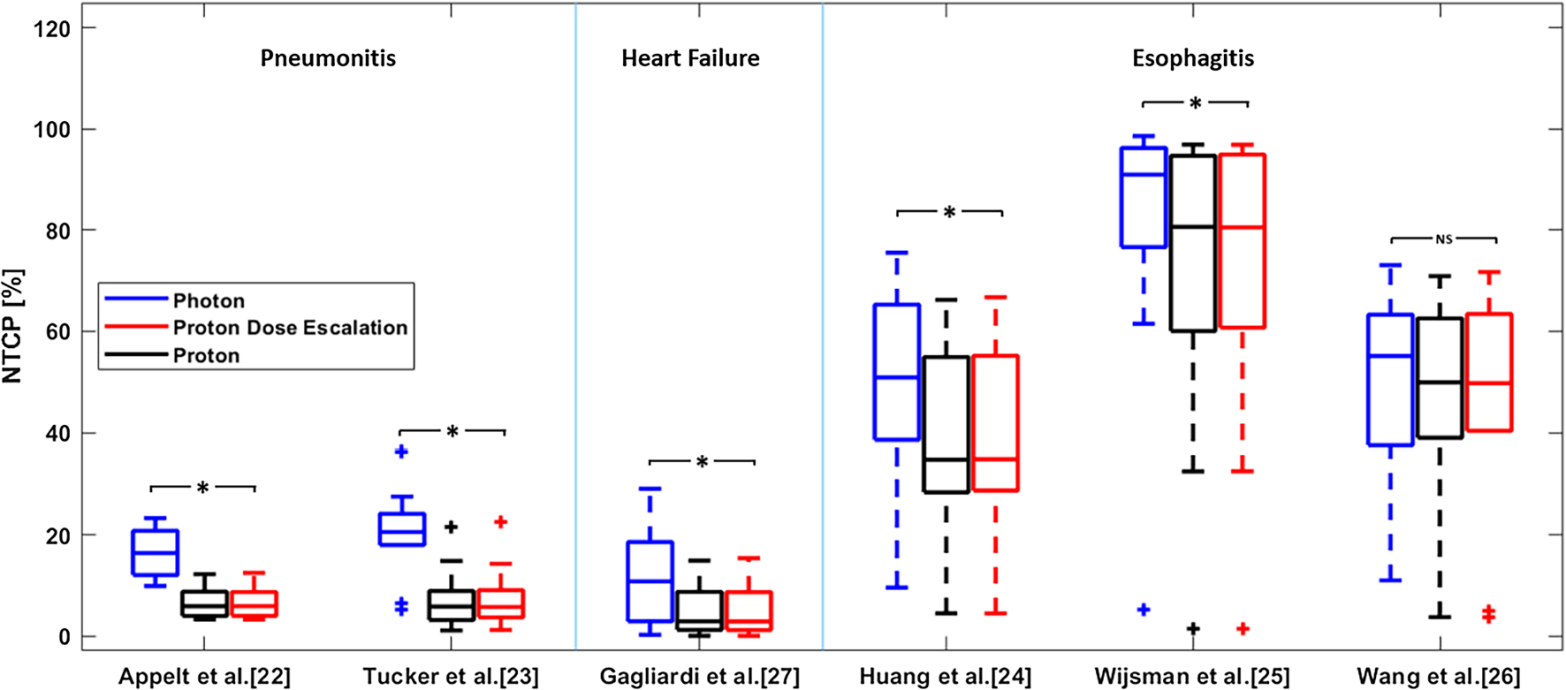
Distribution of NTCP for homogeneous dose prescription photon (blue), proton (black) and dose escalated proton plans (red) of the whole cohort. Significant (Wilcoxon Signed Rank Test) NTCP reductions are observed for the dose-escalated proton plans in lungs, heart and esophagus (excluding the Wang et al. [28] model) compared to the photon plans. Dose escalated did not result in significant increases in NTCP compared to the homogeneous dose prescription proton plans

The use of protons thereby permits dose escalation to a tumor sub-volume with limited to no impact on the surrounding organs, thus maintaining normal tissue benefits compared to photons. Figure 4 shows NTCP’s for non-escalated photon, non-escalated proton and dose escalated proton plans. All NTCP calculations predicted a significant reduction for proton dose escalation plans compared to conventional photon treatments with the exclusion of the Wang et al. [28] radiation-induced esophagitis model, which instead of using the mean esophageal dose, considers the esophagus a more serial organ (n = 0.24, Additional file 1: Supplementary Material S2). Reducing the maximum dose to the esophagus is challenging independent of the modality due to its proximity to the target volume for these patients. On the other hand, the risk of radiation-induced pneumonitis could on average (across all considered models) be reduced by 11.3%, the risk of patient death due to heart failure by 7.4% and the risk for esophagitis reduced by 7.5%.

## Discussion

In this study, we have investigated hypoxia-guided proton dose escalation in a cohort of locally advanced NSCLC patients. Based on our treatment simulations, an image-guided and patient-specific dose escalation by contour strategy allows the mitigation of TCP that may be lost due to the increased radio resistance of hypoxic regions of the tumor. The additional doses to the target were not found to be detrimental to normal tissue sparing, in fact risks of side effects in OARs were significantly lower for proton treatments compared to state-of-the-art photon therapy (Fig. 4).

While it has been shown that increased doses to the target can lead to better tumor control in locally advanced NSCLC [30] patients, the RTOG 0617 trial results [7] have shown that it is essential to balance between escalated dose levels and normal tissue burden. Furthermore, especially at the level of local control there still is a lack of strong clinical evidence that supports the use of PBS proton therapy despite clear dosimetric benefits for critical OARs. While RTOG 1308 will hopefully provide some insight on the photon vs. proton debate for conventional NSCLC treatment, in a scenario where dose escalation is necessary, the improved spatial selectivity of proton therapy could still be leveraged to perform dose-escalation with high precision, while keeping dose to healthy tissue low [31, 32]. As demonstrated in other studies, there is a clear limit to the amount of dose that can be escalated with current state-of-the-art radiotherapy without impacting normal tissue, and proton treatments, taking advantage of lower integral dose, can theoretically push this limit significantly further. OAR sparing is indeed of particular concern in locally advanced NSCLC, as shown by the MDACC trial [33], in which often the primary target is located in the proximity of critical organs such as the heart, lungs or esophagus. Many studies have considered dose escalation before, however mostly focusing on uniformly boosting tumor dose either by an empirical amount or unconstrained escalation until normal tissue constraints are met [15]. In contrast, we have investigated localized and patient-specific dose escalation based on quantitative hypoxia information extracted from PET imaging. This could result in a reduction of excess dose to the tumor and, ultimately, in lower healthy tissue burden. In this study, we complemented the dosimetry evaluation with OER-weighted TCP and NTCP models to estimate the treatment outcome, thus assessing the clinical potential of such anticipated reductions of healthy tissue dose. While the model-based evaluation of dosimetry differences between modalities and treatment techniques cannot replace clinical evidence from clinical trials, it can be used to estimate the potential clinical benefit that can be obtained by using proton therapy against alternative options. For example, a reduction of 10% in the modelled risk of pneumonitis, as observed for most patients in our cohort, would have been sufficient reason to consider proton therapy for these patients according to the Dutch model-based approach for grade 2 complications [34].

In order to tailor the dose prescription to each patient, the latest research results in PET imaging calibration and radiobiological modelling of OER were combined in a practical, but nonetheless patient-specific approach for determining the dose-escalation factor to target variable tumor hypoxia. This method is however affected by a certain degree of uncertainty, starting with the requirement of an accurate measurement of tumor oxygenation in-vivo. The conversion from HX4 PET image intensity to pO_2_-levels has not been extensively studied yet and is therefore possibly prone to errors in absolute oxygen measurements. Furthermore, the higher sensitivity at low oxygen pressures when compared to F-MISO [18] helps discriminating the smallest gradients but also requires utmost accuracy in calibration before use for quantitative dose escalation. In an attempt to minimize the impact of these uncertainties we have defined the dose escalation from the mean SUV in the GTV_hypoxic_ rather than the maximal value. Similarly critical is the radiobiological modelling of OER from the measured tissue hypoxia for which we selected the formulation from Dahle et al.[19]. This parametrization was considered the most appropriate for our study since it is based on a popular model formulation from Wenzl and Wilkens [35] which has been reported to agree well with clinical studies (for photons however), and which was further parametrized on the basis of proton data [19].

The calculation of the DE factor from PET images is possible and, as we have shown in-silico, hypoxia-induced radio-resistance could potentially be overcome using such an approach. Nevertheless, the TCP could not be completely recovered (Fig. 2C). This is due to the chosen dose-escalation-by-contour planning strategy which does not follow the heterogeneous tissue response within the target and the DE definition from the mean intensity of the hypoxia PET images. However, the TCP could be further optimized by taking the median/maximum OER as DE factor if the OAR dose constraints allow or by designing more complex dose escalation strategies with multiple sub-volumes with different levels of dose escalation. As an alternative to dose-painting-by-contour, dose-painting-by-numbers has also been proposed [36, 37] for a finer level of dose adaptation at the image voxel resolution. However, due to the large uncertainties that affect planning, and considering the possibly impaired treatment precision due to breathing motion we consider, at least at the current stage of technology, dose-painting-by-contour to be the more appropriate technique for lung treatments. A further advantage of this method is that standard treatment planning systems can plan for such a dose escalation, obviating the need for more sophisticated optimizers. The dose-escalation strategy presented in this work, i.e. homogeneous dose escalation adapted to the patient-specific hypoxia within a tumor sub-volume, can then be realistically explored in any clinic.

Clearly, another limitation of our study is the fact that the effects of potential organ motion on the treatments have been ignored. Indeed, sensitivity to intra-fractional organ motion is of particular concern in PBS proton therapy due to interplay effect and range uncertainties. In this study, all fields were planned with large PTV margins accounting for the free-breathing target motion, beam angles selected such minimal density gradients are crossed and uniform DE prescriptions obtained using single field (SFO) rather than multiple field optimisation (MFO). Nevertheless, simulations have all been performed on a stationary, mid-ventilation anatomy. This simplification has allowed to investigate the potential of proton therapy in the context of hypoxia dose painting without skewing the results towards a particular planning approach or motion mitigation technique that would likely be modality specific and possibly influenced by the beam delivery performance of a given facility. Necessarily, a discussion has to follow to identify the best treatment approach to preserve localized dose escalation under the condition of motion. As such, our results are presented as a best case scenario and, to some extent, may reflect the experience of treatments in breath hold or with very narrow gating windows. On a similar note, unsafe dose-escalation can indeed be harmful, and therefore image guidance and motion mitigation techniques may need to be re-evaluated taking into account the specific requirement of dose painting, beyond the current photon vs. proton debate and trials. The technical realization however, including appropriate margins and motion mitigation to bring these benefits into fruition, goes beyond the present discussion.

Finally, we have compared dose distributions and corresponding NTCPs of dose-escalated proton plans with non-escalated uniform dose VMAT plans to evaluate proton therapy against state-of-the-art photon treatments at the level of normal tissue toxicity. Dose escalation with VMAT was not part of the detailed investigations, since photon planning for advanced stage NSCLC patients with large tumors in proximity to the mediastinum is already challenging for a prescription dose of 70 Gy, if normal tissue dose constraints are not to be violated, especially those for lung tissue. The mean DVHs for the VMAT plans in Fig. 3 show how for most plans the OAR constraints (Lung V_20Gy_ < 37% and V_5Gy_ < 60%) were barely fulfilled across the cohort. Escalating the dose to a tumor sub-volume with that modality would inevitably increase doses to the surrounding organs and thus compromise constraints. Moreover, in order to assess the toxicity that would potentially be associated with proton treatments, considering the most aggressive plans, i.e. including dose escalation, is the most sensible choice.

## Conclusions

We presented a clinical option to target patient-specific tumor hypoxia with dose escalation. In a cohort of ten patients with stage IIb-IIIc NSCLC, median partial oxygen pressures in the most hypoxic region within the primary target ranged from 5 to 9.2 mmHg, leading to an overestimation of radiotherapy efficacy by up to 10% of TCP. Local control was effectively recovered on average within 0.9% of nominal TCP with a patient-specific dose escalation up to 22% of the prescribed dose. The increased sparing of normal tissues using proton therapy has the potential for such biologically guided treatments compared to the latest generation of photon treatments, reducing the risk of radiation-induced pneumonitis (11.3%), patient death due to heart failure (7.4%) and esophagitis (7.4%).

## Supplementary Information

**Additional file 1: Supplementary Material S1.** Treatment planning constraints as in RTOG 1308. **Supplementary Material S2.** Detailed description of TCP and NTCP models. **Supplementary Material S3.** Example dose distributions for photon and proton plans. **Supplementary Material S4.** Example LET distributions from proton plans.

## Data Availability

The data presented in this study are available from the senior author (G.F.) or first author (A.K.) upon reasonable request.

## Abbreviations

CT: Computed Tomography
CTV: Clinical Target Volume
DE: Dose Escalation Factor
DVH: Dose-Volume Histogram
EUD: Equivalent Uniform Dose
FDG: Fluorodeoxyglucose
F-FAZA: Fluoroazomycin arabinoside
F-HX4: Flortanidazole
F-MISO: Fluoromisonidazole
GTV: Gross Tumor Volume
GTV_hypoxic_: Gross Tumor Volume with hypoxia
LET_d_: Dose-Averaged Linear Energy Transfer
LKB: Lyman-Kutcher-Burman Model
NSCLC: Non-Small Cell Lung Cancer
NTCP: Normal Tissue Complication Probability
OAR: Organ-At-Risk
OER: Oxygen Enhancement Ratio
PBS: Pencil Beam Scanning
PBSPT: Pencil Beam Scanning Proton Therapy
PET: Positron Emission Tomography
pO_2_: Partial Oxygen Pressure
PTV: Planning Target Volume
RBE: Relative Biological Effectiveness
RTOG: Radiation Therapy Oncology Group
SFO: Single Field Optimization
SUV: Standardized Uptake Value
TCP: Tumour Control Probability
UICC: Union for International Cancer Control
VMAT: Volumetric Modulated Arc Therapy
