# Supplementary material for: Investigating the potential of proton therapy for hypoxia-targeted dose escalation in non-small cell lung cancer
Source: Radiat Oncol. 2021 Oct 11;16:199. doi: 10.1186/s13014-021-01914-2 (PMC8507157; doi:10.1186/s13014-021-01914-2)

**8. Supplement**

***S1. Treatment planning constraints as in RTOG 1308***

S1: Target and organ constraints employed in the treatment planning process as specified in the ongoing RTOG 1308 randomized trial. MLD: Mean lung dose. V_x_ is the volume of a structure that is given at least x Gy_RBE_. D_x_ represents the dose given to a volume of at least x cc.

| **Target volume/ Organ at risk** | **Primary Constraints** | **Acceptable Deviation** |
| --- | --- | --- |
| **CTV** | V_70 Gy_ = 100 % | V_70 Gy_ > 99 % |
| **PTV** | V_70 Gy_ = 95 % V_84 Gy_ < 0.03 cc  D_0.03 cc_ > 59.5 Gy | V_66.5 Gy_ = 95 %  V_87.5 Gy_ < 0.03 cc  D_0.03 cc_ > 52.5 Gy |
| **Lungs-GTV** | V_20 Gy_ < 37 %  MLD < 20 Gy  V_5 Gy_ < 60% | V_20 Gy_ < 40 % or  MLD < 22 Gy  V_5 Gy_ < 65% |
| **Heart** | V_30 Gy_ < 50 %  V_45 Gy_ < 35 % | V_30 Gy_ < 55 %  V_45 Gy_ < 40 % |
| **Spinal Cord** | V_50 Gy_ < 0.03 cc | V_52 Gy_ < 0.03 cc |
| **Esophagus** | V_74 Gy_ < 1 cc | V_74 Gy_ < 1.5 cc |
| **Brachial Plexus** | V_70 Gy_ < 3.0 cc  V_74 Gy_ < 1.0 cc  V_75 Gy_ < 0.5 cc | V_75 Gy_ > 0.5 cc |

***S2. Model selection***

***S3. Example Dose Distributions***

Patient 2

S2: Example dose distributions for the photon, proton and proton dose escalation plans for patients 02,06 and 10. Proton field directions are indicated by the arrows.


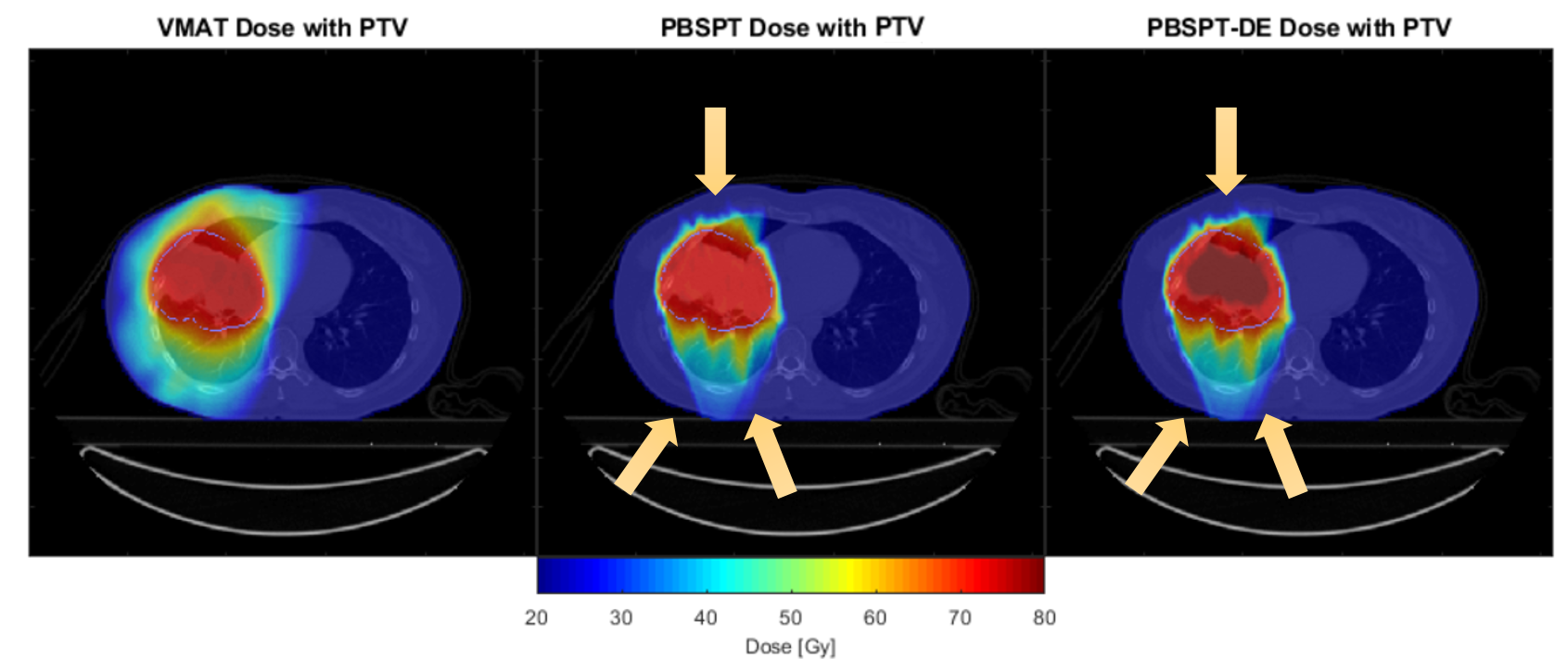


Patient 6


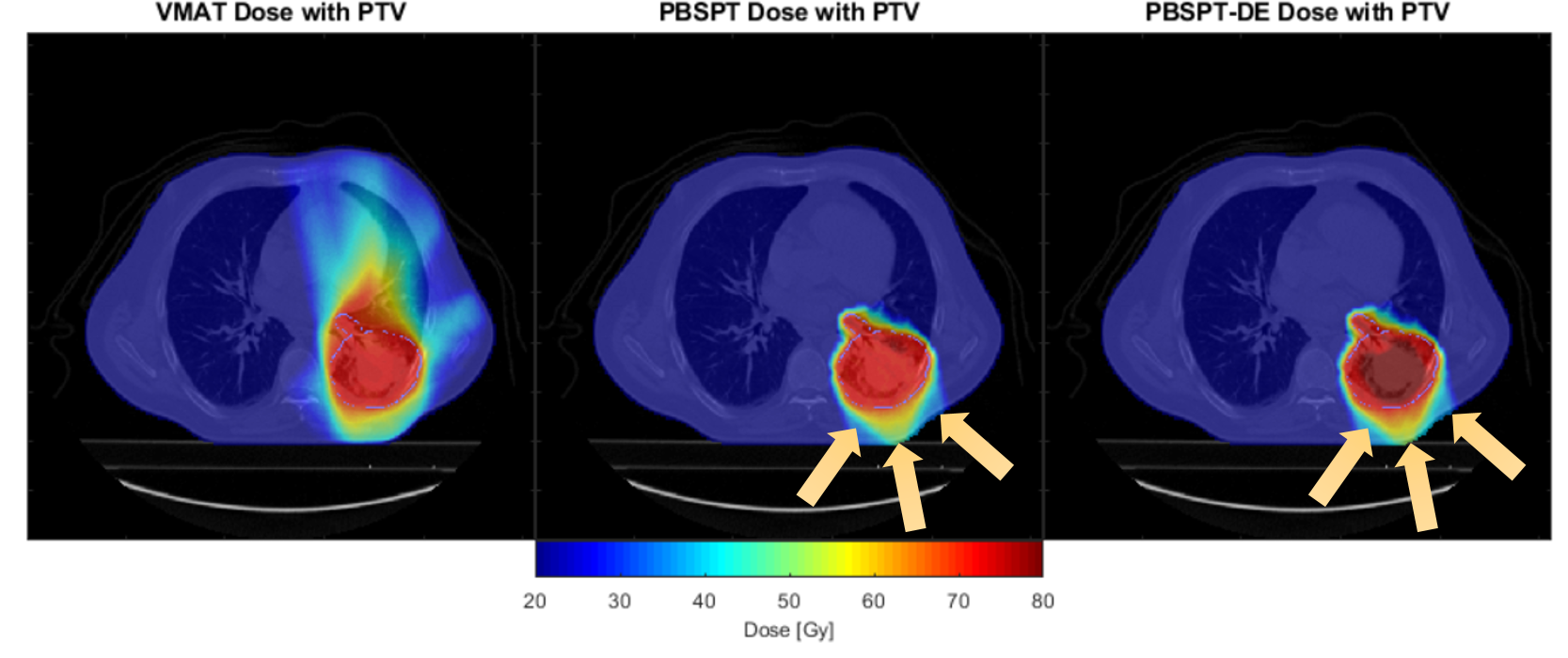


Patient 10


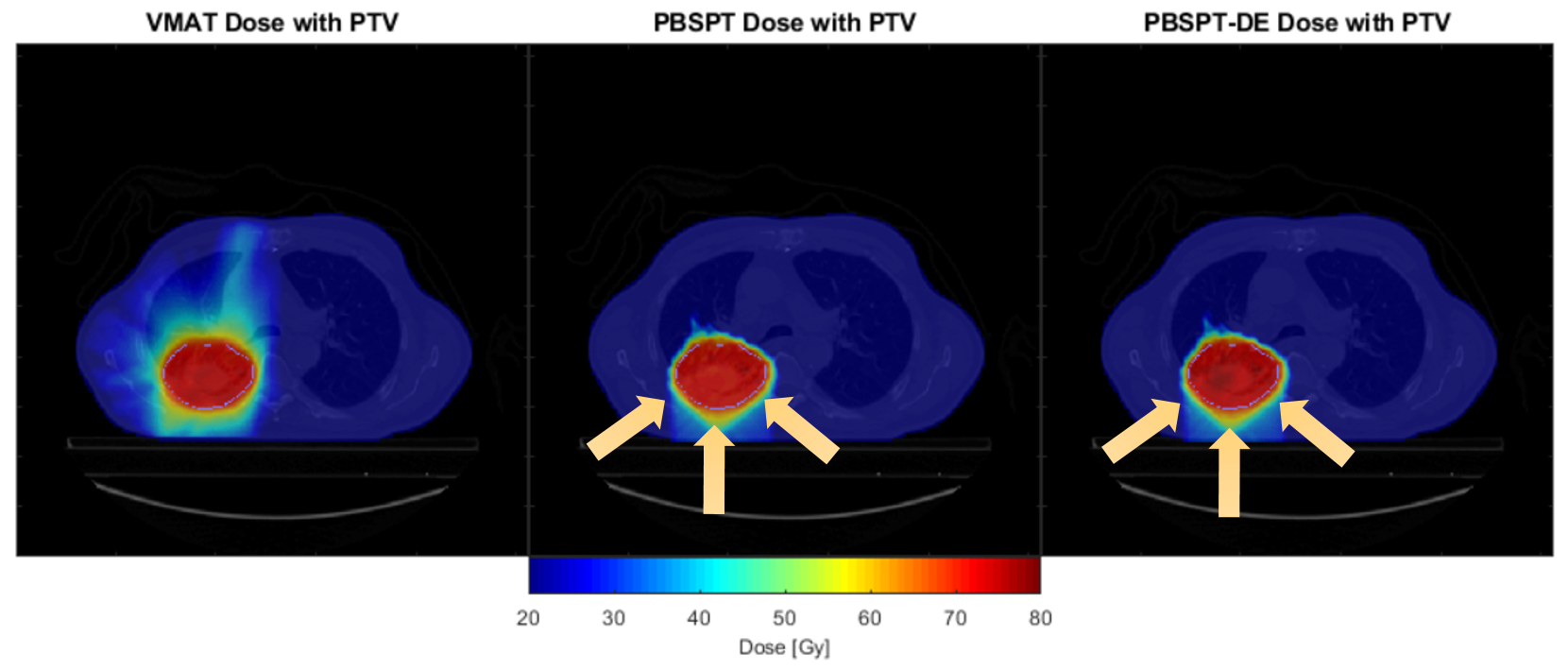


***S4. Example LET Distributions***

S4: Exemplary LET Distributions for patients 02 and 06 (left panels). LET in the PTV (red contour) is relatively constant (between 2-3 keV/µm). The right panels show LET differences between the conventional proton plans and the dose escalated ones. Differences are minor (< 0.5 keV/µm), but a slight increase in LET can be observed due to the dose escalation to the hypoxic subvolume (black contour). Proton field directions are indicated with the black arrows. Dose distributions for these patients can be found in S3.


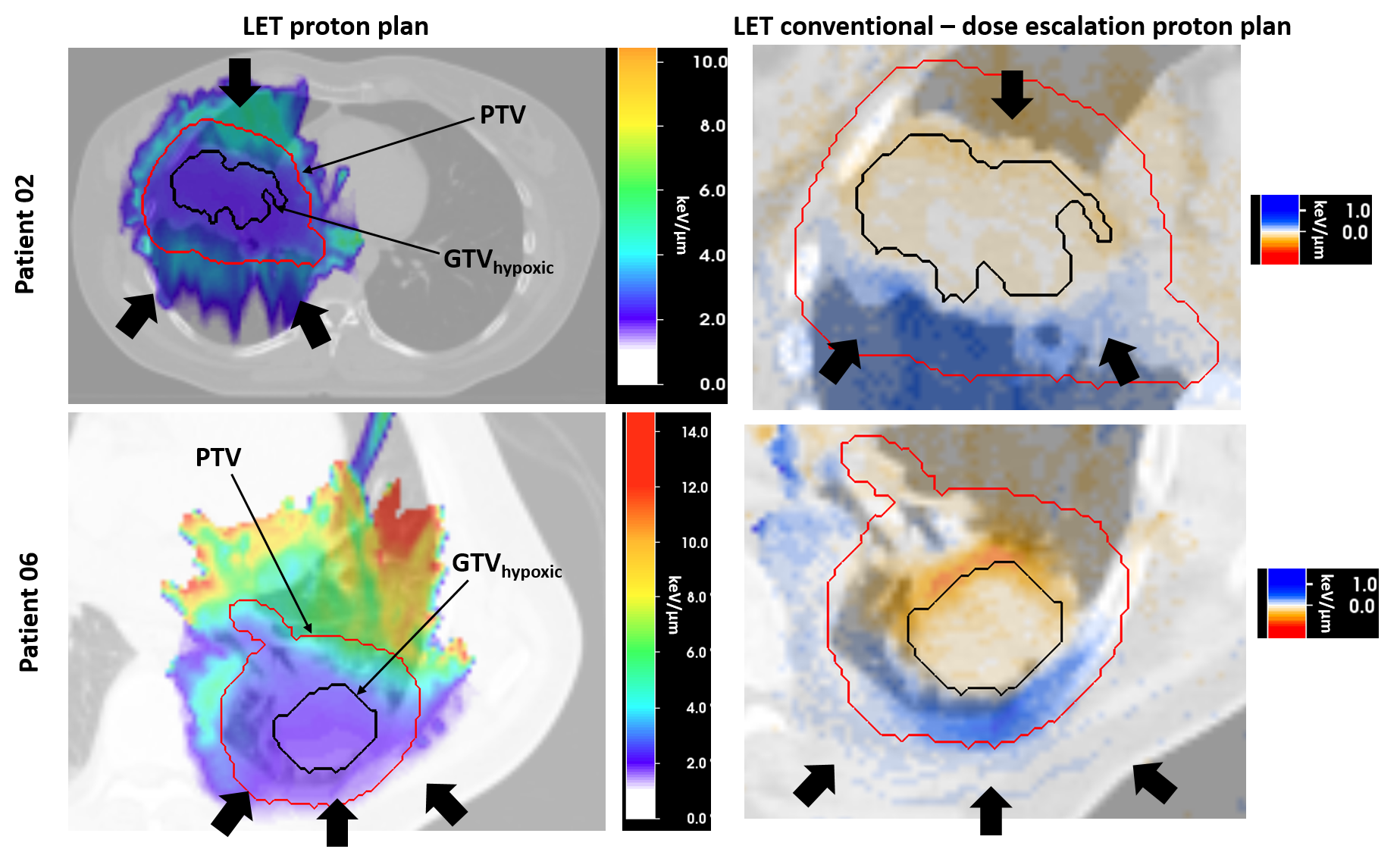

Supplement: Supplementary file 1 — Additional file 1: Supplementary Material S1. Treatment planning constraints as in RTOG 1308. Supplementary Material S2. Detailed description of TCP and NTCP models. Supplementary Material S3. Example dose distributions for photon and proton plans. Supplementary Material S4. Example LET distributions from proton plans. [file 13014_2021_1914_MOESM1_ESM.docx]
